# Homo- and Copolymer Hydrogels Based on N-Vinylformamide: An Investigation of the Impact of Water Structure on Controlled Release

**DOI:** 10.3390/gels9040333

**Published:** 2023-04-14

**Authors:** Maytinee Yooyod, Sukunya Ross, Premchirakorn Phewchan, Jinjutha Daengmankhong, Thanyaporn Pinthong, Nantaprapa Tuancharoensri, Sararat Mahasaranon, Jarupa Viyoch, Gareth M. Ross

**Affiliations:** 1 Biopolymer Group, Department of Chemistry, Faculty of Science, Naresuan University, Phitsanulok 65000, Thailand; 2 Biopolymer Group, Department of Chemistry, Center of Excellence in Biomaterials, Faculty of Science, Naresuan University, Phitsanulok 65000, Thailand; 3 Department of Pharmaceutical Technology, Faculty of Pharmaceutical Sciences, Naresuan University, Phitsanulok 65000, Thailand; 4 Department of Pharmaceutical Technology, Faculty of Pharmaceutical Sciences, Center of Excellence for Innovation in Chemistry, Naresuan University, Phitsanulok 65000, Thailand

**Keywords:** N-vinylformamide, hydrogels, controlled release, water structure, N-hydroxyethyl acrylamide, 2-carboxyethyl acrylate, UVLED photopolymerization

## Abstract

This study investigated the performance of novel hydrogels based on poly (N-vinylformamide) (PNVF), copolymers of NVF with N-hydroxyethyl acrylamide (HEA) (P(NVF-co-HEA)), and 2-carboxyethyl acrylate (CEA) (P(NVF-co-CEA)), which were synthesized by photopolymerization using a UVLED light source. The hydrogels were analyzed for important properties such as equilibrium water content (%EWC), contact angle, freezing and non-freezing water, and diffusion-based in vitro release. The results showed that PNVF had an extremely high %EWC of 94.57%, while a decreasing NVF content in the copolymer hydrogels led to a decrease in water content with a linear relationship with HEA or CEA content. Water structuring in the hydrogels showed appreciably more variance, with ratios of free to bound water differing from 16.7:1 (NVF) to 1.3:1 (CEA), corresponding to PNVF having ~67 water molecules per repeat unit. The release studies of different dye molecules followed Higuchi’s model, with the amount of dye released from the hydrogels depending on the amount of free water and the structural interactions between the polymer and the molecule being released. The results suggest that PNVF copolymer hydrogels have potential for controlled drug delivery by altering the polymer composition to govern the amount and ratio of free to bound water contained in the hydrogels.

## 1. Introduction

Polymer materials remain a popular choice for controlled release systems, having garnered significant attention and being utilized in numerous domains, such as pharmaceuticals, medicine, agriculture, and material science. These materials include polymeric lyotropic liquid crystal (e.g., micelles [1] and cubosome [2]), capsules [3], nanofibers [4,5], and hydrogels [6]. Hydrogel systems are particularly suited for controlled release thanks to their advantageous inherent properties, such as high water contents and tailored mechanical strength. The water present enables simple loading methods for water-soluble drug molecules, such as passive diffusion. There has been a growing interest in analyzing the structure of water within hydrogels, as it is becoming increasingly apparent that it plays a crucial role in numerous applications. For example, in the transport of small molecules, water mobility plays a crucial role, with the water state affecting the pharmacokinetics such as drug solubility, absorption/desorption, diffusion, and release [7,8,9].

Generally, it has been observed that the transport and release of molecules can be primarily attributed to the freezing of water within the polymer matrix [10]. Nonetheless, the impact of the adsorbed water state on the kinetics of drug release still requires further research attention. The concept of water states provides an initial understanding of molecular interactions between water molecules and polymers in hydrogels. Specifically, water can exist as free water, intermediate water, or bound water, with the latter category encompassing water that is physically bonded to polymer chains through strong hydrogen bonds. During the initial absorption of water by a dry hydrogel, polar and hydrophilic groups become hydrated, resulting in the formation of “primary bound water”. As the network swells, hydrophobic groups become exposed, leading to the formation of “secondary bound water”. These two types of bound water are collectively known as “total bound water” [11]. After the ionic, polar, and hydrophobic sites become saturated with bound water, the hydrogel will imbibe additional water owing to the osmotic driving force of the network chains towards infinite dilution, resulting in the formation of “free water” or “bulk water”. This additional swelling water fills the space between the network chains and/or the center of larger pores, macropores, or voids, until an equilibrium swelling level is reached [12].

In hydrogel synthesis, the selected monomers govern the water content and water structuring and, therefore, the performance in the desired application. In this work, N-vinylformamide (NVF) was selected as the main monomer. N-vinylformamide (NVF) is an isomer of acrylamide (AAm), but possesses some favorable properties over AAm such as lower toxicity [13], more hydrophilicity [14], more reactivity [15], and being liquid at room temperature, and thus is easier to use [16]. The physical properties of poly(N-vinylformamide) (PNVF) gels are quite like the technologically important hydrogels of polyacrylamide (PAAm) that are used in electrophoresis, chromatography, cosmetics, biomedical implants, superabsorbent products, and soil conditioners, among numerous other applications. PNVF gels are also chemically related to poly(N-vinylpyrrolidone) (PNVP) gels, another important biomedical hydrogel, widely used in contact lenses, drug delivery systems, and wound dressings. While PNVF has the capability of creating intriguing hydrogels, there are concerns about its mechanical performance in certain applications. Thus, incorporating NVF with other co-monomers through copolymerization can help regulate the ultimate performance of the resulting hydrogels.

N-hydroxyethyl acrylamide (HEA) is an interesting commercially available monomer, containing two hydrophilic groups, a hydroxyl group and an amide group, separated by two methylene groups. Poly(N-hydroxyethyl acrylamide) (PHEA) can serve as a promising biomaterial with long-term antifouling and durability suitable for biomedical applications [17,18,19,20]. Recently, Chen et al. [21] found that PHEA could self-crosslink to form a physical gel without any chemical crosslinkers during polymerization, with a critical gelation concentration of as low as 3 wt% HEA content. However, the pure physical crosslinked PHEA gel was relatively weak when compared with chemically crosslinked gels [22]. 2-carboxyethyl acrylate (CEA) is a high-molecular-weight vinyl acid monomer when compared with acrylic acid (AA) and methacrylic acid (MAA) (144 g/mol vs. 72 and 86 g/mol, respectively). CEA is classified as a low toxicity, water-soluble monomer that has been shown to modify the rheological properties of ceramic suspensions and minimize the negative effect of oxygen inhibition [23]. Its molecular structure, which includes a carboxyl group, is expected to have interesting properties with different water/monomer distribution characteristics from the more traditional AA and MAA functional monomers. Additionally, CEA contains an ester group, making it a vinyl acid monomer with two carbonyl groups, providing a direct molecular comparison to functional vinyl monomers with −COOH and −OH end groups [24]. The copolymerization of HEA and CEA with NVF has the ability to display interesting behavior. It also provides additional mechanical reinforcement, which is particularly noticeable in the case of HEA.

In this work, copolymers of NVF with HEA (P(NVF-co-HEA)) and CEA (P(NVF-co-CEA)) were synthesized and chemically crosslinked with a divinyl crosslinker at the same time to form hydrogels. This used conventional free radical polymerization utilizing a photoinitator and the latest UVLED light sources, which provide a simple, cost-effective, rapid fabrication method. All hydrogel compositions were fully characterized using a number of techniques such as surface hydrophilicity, total water content (%EWC), and water structure reported as the amounts and ratio of freezing (free) and non-freezing (bound) water. A diffusion-based in vitro release study was used to determine the release profiles and kinetics of three distinct organic molecules (orange II sodium salt, crystal violet, and Congo red) from the hydrogels. These dye molecules have varying structures, functional groups, molecular weights, and partition coefficients, making them effective for predicting the release behavior of other organic species from the hydrogels owing to their simplicity and effectiveness as a test [25]. Overall, this work successfully synthesized and characterized hydrogels using a simple and cost-effective method and evaluated their release behavior using three distinct organic molecules as a model system.

## 2. Results and Discussion

When the P(NVF-co-HEA) and P(NVF-co-CEA) hydrogels are synthesized without water, they are stiff and brittle materials, but, after soaking in water for 5 days, they become soft and flexible gels. The pliability of the hydrogels was contingent upon the type and proportion of copolymer utilized, as evidenced by the increased flexibility and swelling of the hydrogels when HEA was incorporated in comparison with CEA. The properties of the fabricated hydrogels were characterized throughout this investigation.

### 2.1. Hydrophilicity and Water Properties

**Figure 1:**
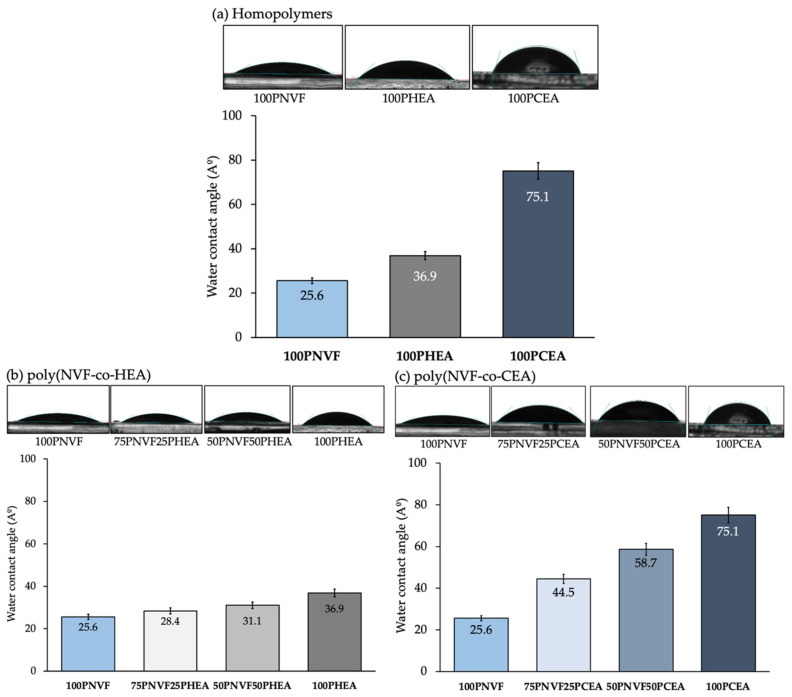
Contact angle of the homopolymer hydrogels (**a**) and PNVF-copolymer hydrogel series: (**b**) P(NVF-co-HEA) and (**c**) P(NVF-co-CEA). The corresponding images of water droplets are depicted for each composition.

The goal of this work lies in understanding how we can influence the delivery of active agents by altering the copolymer composition of the NVF-based hydrogel system. At the outset, we evaluated the uniformity and replicability of the hydrogel samples prepared by measuring their dimensions and weight. To achieve this, we employed an 11 mm diameter cork borer to cut each hydrogel sample for characterization purposes. Table S1 presents all the data, indicating that the hydrogels exhibit remarkable consistency across all samples, with thickness ranging between 0.61 and 0.70 mm and weights varying from 0.84 to 0.132 g. Notably, PCEA had the lowest values, while PNVF had the highest. Following this, we gauged the hydrophilicity of the hydrogels by measuring the water contact angle of the samples. Figure 1 presents the contact angle of each sample from Table 1. The contact angle of the homopolymer hydrogels showed that 100PNVF, 100PHEA, and 100PCEA were 25.6°, 36.9°, and 75.1°, respectively. Importantly, all of these values are under 90° and, therefore, all the hydrogel samples are defined as hydrophilic. The contact angles of copolymers varied as the ratio of copolymer was altered. This followed a near linear relationship between the two monomers used. For example, as PNVF has the lowest angle among the three polymers, when the ratio of either PHEA or PCEA is increased, the contact angle of the gel trends towards the higher angle. This is typical behavior for materials of this type as the surface properties are directly linked to the functional groups present at the surface of the hydrogel in the hydrated state.

Upon verifying that the hydrogels exhibited hydrophilic properties, we proceeded to quantify the amount of water they contained, typically expressed as a percentage of equilibrium or equilibrium water content (%EWC). This metric holds significant significance as it is frequently indicative of the hydrogels’ performance in their intended use. However, how the water interacts with the polymer chains or water structuring (water states) is also vital to fully understand and predict how the gels will perform. Therefore, the equilibrium water content (%EWC) and water states in the hydrogels were assessed using the methods described in Section 4.3 and Section 4.4.

**Figure 2:**
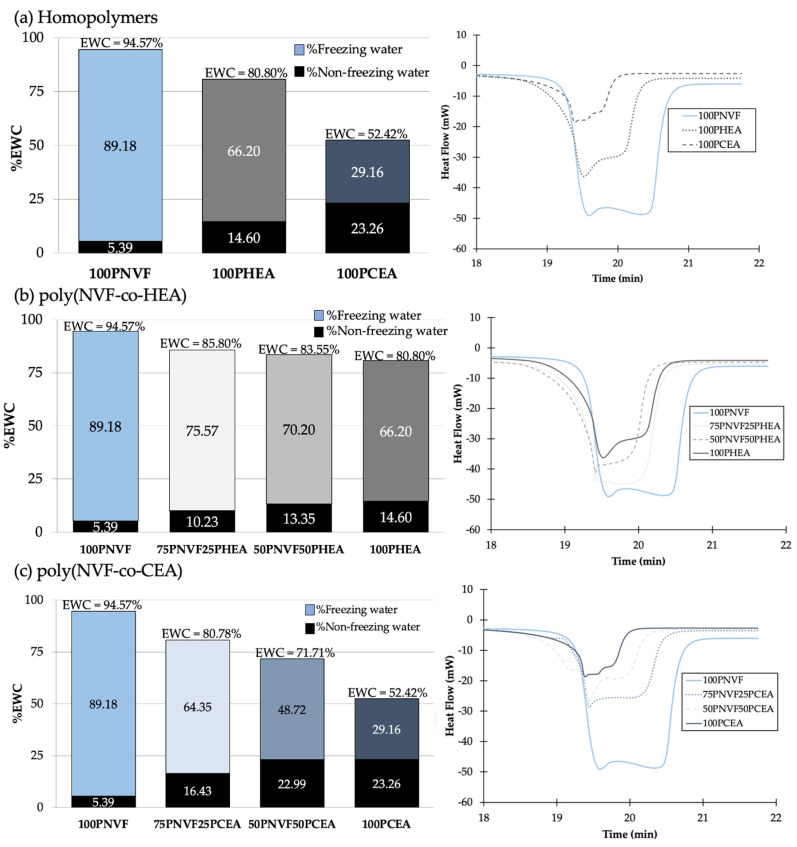
%EWC (**left**) and DSC endotherms (**right**) of (**a**) homopolymers, (**b**) P(NVF−co−HEA) hydrogels, and (**c**) P(NVF−co−CEA) hydrogels.

The water contents (%EWC) of each sample were observed and are shown in Figure 2 (left). The %EWC of homopolymer hydrogels (Figure 2a) showed that 100PNVF, 100PHEA, and 100PCEA were 94.57%, 80.80%, and 52.42%, respectively. When the copolymers of P(NVF-co-HEA) (Figure 2b) and P(NVF-co-CEA) (Figure 2c) were measured, the %EWC presented a similar trend to that seen with the contact angle, with a close to linear relationship between %EWC and the content of the copolymer (trending towards the lower homopolymers %EWC). In both examples, PHEA and PCEA have a lower %EWC than PNVF; therefore, the trend is towards a decrease in the %EWC in the copolymer hydrogel samples.

**Figure 3:**
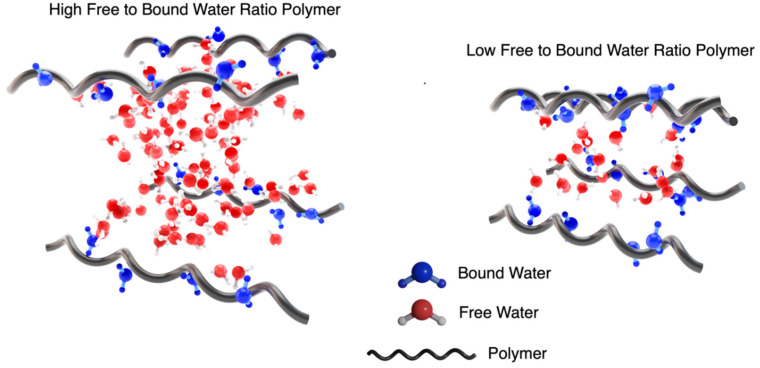
Schematic representation of the free to bound water in a high ratio and low ratio system.

Each %EWC bar is further split to show the percentage of the freezing and non-freezing water present in each hydrogel. These are calculated from the DSC isotherms (Figure 2 (right)), with the area under the peak related to the amount of freezing water present in the hydrogel. Although these values are presented as a percentage, it is possible to calculate the number of water molecules per repeat unit. This is calculated using the mass of the repeat unit and the amount of water present in the hydrated gel. For PNVF, the number of water molecules per repeat unit is ~67, while for PHEA and PCEA, the numbers are ~27 and ~9, respectively. This highlights the large amount of water associated with the NVF polymer chains in the hydrogel, with multiple hydration shells and water between the crosslinks. Another interesting property is the ratio between the number bound and free water molecules in the gels. For the homopolymer samples, the ratios of free to bound water are PNVF—16.7:1, PHEA—4.5:1, and PCEA—1.3:1 (Table 2). Figure 3 displays a schematic representation of these findings. The polymer with a high free to bound water ratio has considerably more water molecules between each of the polymer chains, which results in a ‘looser’ hydrogel network, while also allowing for more water to solubilize other molecules and hold them in the hydrogel matrix. For the polymer with a low free to bound water ratio, the reverse is observed, with a ‘tighter’ network with polymer chains closer together and substantially less water molecules available.

For the copolymers of P(NVF−co−HEA) and P(NVF−co−CEA), the ratios of free to bound water are also presented in Table 2. It is evident from table that, even with a small variation in %EWC, there is a larger variation in the ratios of free to bound water. For example, when water contents change from 80 to 85%EWC, the free to bound ratio can double and vary from ~4 to 8. The work of Zentner et al. [10] stated that the freezing or free water in hydrogels is the key factor in transport and release in polymer matrixes. Therefore, the range of free water ratios in these sample allows for further understanding of the release behavior.

### 2.2. Transport and Release Properties

To assess the transport and release of these hydrogels, a series of diffusion-based control drug release studies were conducted, wherein each hydrogel was soaked in a dye solution of a known concentration (0.0001 M) for 96 h. Three distinct dyes were used (orange II sodium salt, crystal violet, and Congo red) with different functional groups, molecular weights, and n-octanol-water coefficients (log *p*). Table 3 summarizes the differences between the dyes, with the molecular weight ranging from ~350 to 697 and log *p* varying from −0.95 to 2.63. Although, one similarity was that all of the selected dyes were water soluble. The cumulative amount of dye released was calculated from standard calibration curves of each dye (Figure S1), with the amount of dye release recorded as a dose fraction. To evaluate the uptake, the UV absorption of dye solution was measured both before and after uptake. The homopolymer hydrogels loaded different amounts of the available dye for PNVF, PHEA, and PCEA, with O2S having loading amounts of 19.1%, 2.5%, and 13.8%, respectively. For CV, the loaded amounts were 94.6%, 84.3%, and 99.5%, respectively, while for CR, the amounts were 35.8%, 27.2%, and 12.0%, respectively. These findings indicate that CV was the easiest dye to incorporate into the hydrogel matrices. The uptake of CR followed the trends observed in EWC and free water contents, whereas for O2S, PHEA exhibited minimal uptake.

**Figure 4:**
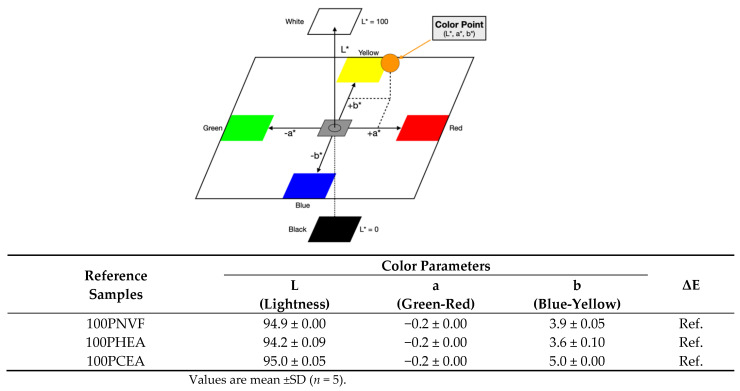
Color parameter measurements are based on the three-dimensional CIE color space; the table shows the total color difference parameter of reference homopolymer hydrogels.

**Figure 5:**
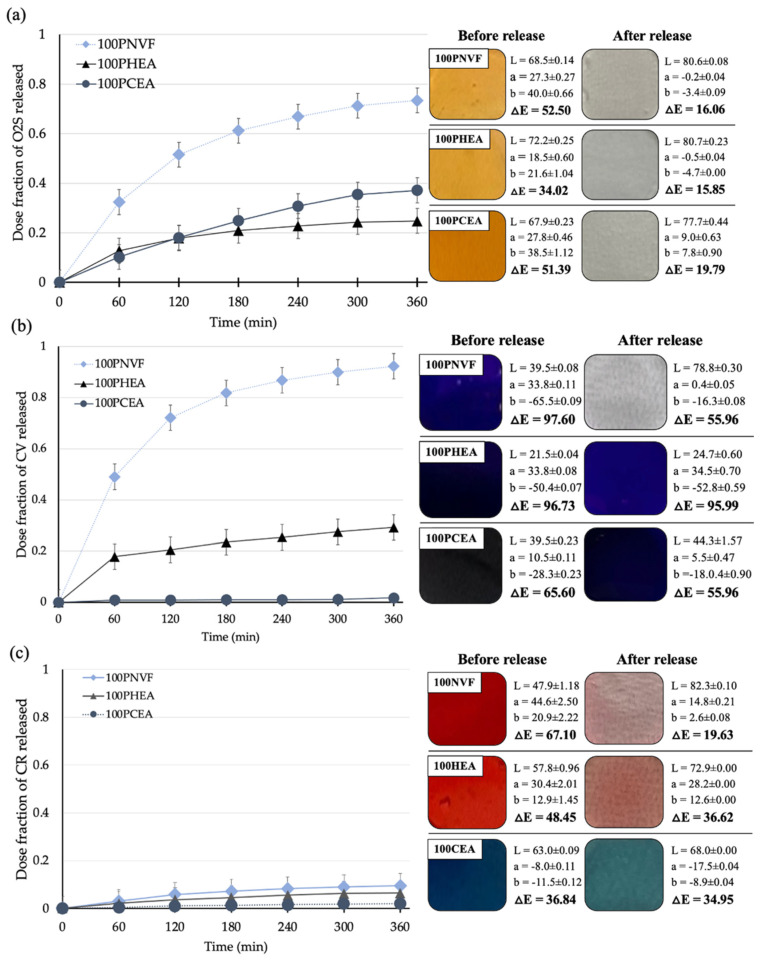
Dye release profiles of homopolymer—100PNVF, 100PHEA, and 100PCEA (**left**) and visual color and total color difference parameters of hydrogels (**right**) before and after dye release: (**a**) orange II sodium salt, (**b**) crystal violet, and (**c**) Congo red.

In order to first assess the hydrogels’ release, the color parameters of the hydrogels were measured before and after dye release and are presented along with the dye release profiles. For the homopolymer hydrogel samples, the color parameters, consisting of L, a, and b, are presented in Figure 4. The ΔE values are used here as reference numbers (Ref.) before uptake and release of the dyes. ΔE is measured on a scale from 0 to 100, where 0 is no color difference and 100 indicates complete distortion. Figure 5 shows that, when the three dyes are up taken into the hydrogels, the color of each gel changes considerably.

The cumulative amount of dye released from the homopolymer hydrogel samples was measured using the method presented in Section 4.6, with the release in DI water shown in Figure 5 (left). The release profiles show that 100PNVF releases the largest amount of dye when compared with the other polymers for each of the three dyes. This is in keeping with the theory that the polymer matrix containing the highest free water content has the ability for the greatest release. This is owing to potentially having the largest reservoir of dye and the fact that the dye that is incorporated into the gel should also be fully solubilized by the free water and thus available for release. In direct contrast to this, 100PCEA gives the lowest release across the range of dyes. Although, PCEA presents the largest differences in terms of both the release profiles and the color of gels before and after release. This may be because PCEA contains the carboxyl pendant group, thus certain dyes can be strongly held in the hydrogel structure by interacting with the polymer structure. This also lowers the pH of the hydrogel, which was confirmed by taking a litmus paper strip and placing it on the surface of the hydrogel with a pH value of 4.5.

Optical photographs and the color parameters in Figure 5 (right) visually allow for the amount of dye released to be tracked. The change in color parameter of the homopolymer with the different dyes shows that the value of each parameter varies both before and after releasing. The 100PNVF hydrogels presented the largest change in the total color difference parameter (ΔE) for each dye. Thus, the release confirmed the cumulative release data in that the PNVF hydrogels released the most dye over the tested period. Again, we see that the PCEA gels show considerable differences when compared with the other gels in terms of the physical color of the gel as well as the color parameter; that is, from the lower pH of the dye/gel combination transitioning into new color, which is most evident with Congo red, which has a color transition at a certain pH in this region. Congo red gives a red color at pH > 5.0 and a blue color at pH < 3.0 [26].

**Figure 6:**
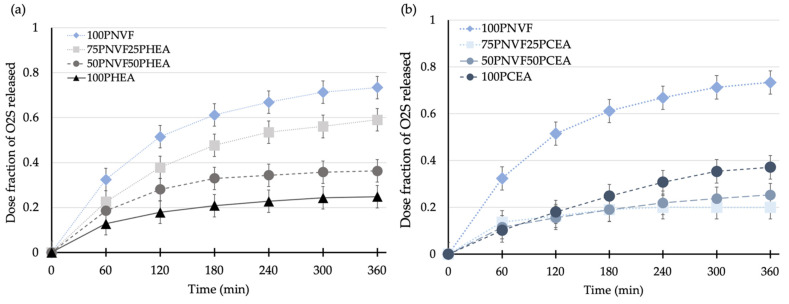
Release profiles of (**a**) P(NVF-co-HEA) hydrogel series and (**b**) P(NVF-co-CEA) hydrogel series with 0.0001 M orange II sodium salt dye solution.

**Figure 7:**
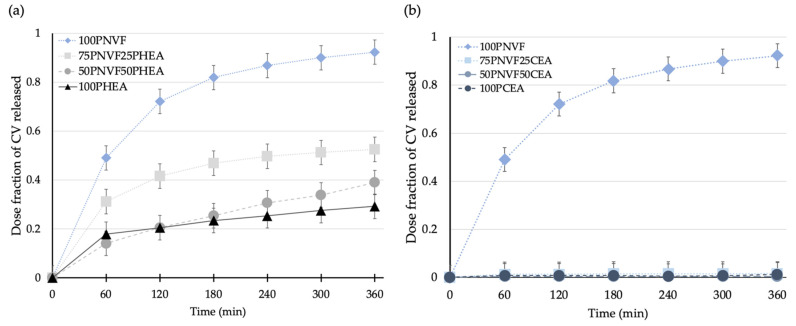
Release profiles of (**a**) P(NVF-co-HEA) hydrogel series and (**b**) P(NVF-co-CEA) hydrogel series with 0.0001 M crystal violet solution.

**Figure 8:**
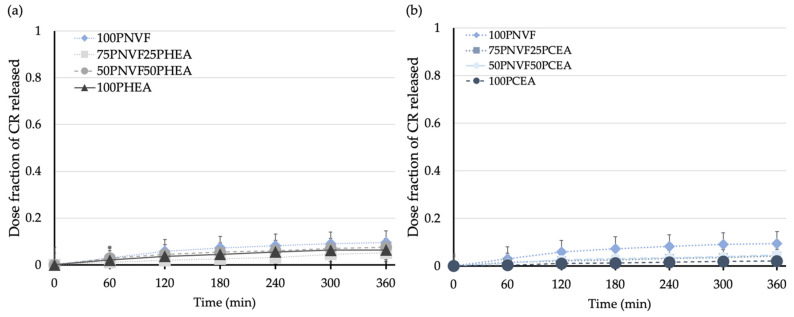
Release profiles of (**a**) NVF and HEA hydrogel series and (**b**) NVF and CEA hydrogel series with 0.0001 M Congo red solution.

The next step was to measure the cumulative release of the copolymer hydrogels. Figure 6, Figure 7 and Figure 8 present the release profiles of P(NVF–co–HEA) and P(NVF–co–CEA) copolymer gels with the three selected dyes (O2S, CV, and CR). Figure 6 displays the release profiles of the copolymer hydrogel samples after the uptake of orange II sodium salt (O2S) dye. The results show that, as the amount of either HEA or CEA is increased (25 or 50wt%), the amount of O2S released decreases. A noteworthy observation is that 100PCEA gives a higher value than the copolymers. The values are similar for the copolymer gels and, because CEA is acidic, it affects the color of the gel, meaning that errors associated with this pair are higher than most of the other samples. For the P(NVF–co–HEA) samples, we find a reduction in the release profile curves as the composition of the copolymer changes.

Figure 7 shows the release profiles of the copolymer hydrogels after the uptake of crystal violet dye. The differences in the release between P(NVF-co-HEA) and P(NVF-co-CEA) are the most pronounced of all of the dyes tested. The main reason for this is that PCEA contains carboxylate groups, which can strongly interact with the CV structure, whereas PHEA contains hydroxyl groups, which can interact with the amide groups of CV, but not as intensely. Thus, from the color pictures in Figure 6b, we can see that both PHEA and PCEA present a dark blue/purple color, but with CEA being darker after uptake and remaining almost the same color after release, where the HEA color is less intense.

Figure 8 shows the release profiles of the copolymer hydrogels after the uptake of CR and the results show that CR gives the lowest release of all of the tested dyes. From Figure 5, we see that none of the homopolymers fully released CR during the 6 h release period. The release profiles, although low, are also the most linear; although the NVF homopolymer still releases the most dye, the remaining samples release very similar amounts, with the exact order not clearly defined. Both of these findings are the result of the inherent properties of CR, which contains both sulfonic acid and amine groups, and thus has the ability to interact with all three of the polymers present in the different gels.

Most of the release data shown (Figure 5, Figure 6, Figure 7 and Figure 8) exhibit non-linearity with respect to time, indicating a decreasing release rate tendency over time. To better understand the release kinetics, three release kinetic models were used to assess cumulative release: zero-order (cumulative release vs. time), first-order (log cumulative release vs. time), and Higuchi’s (cumulative release vs. square root of time). Higuchi’s model is typically used for diffusion-controlled release systems that contain dispersed active ingredients within a polymeric matrix, and it has been confirmed by other researchers as valid [27,28,29].

**Figure 9:**
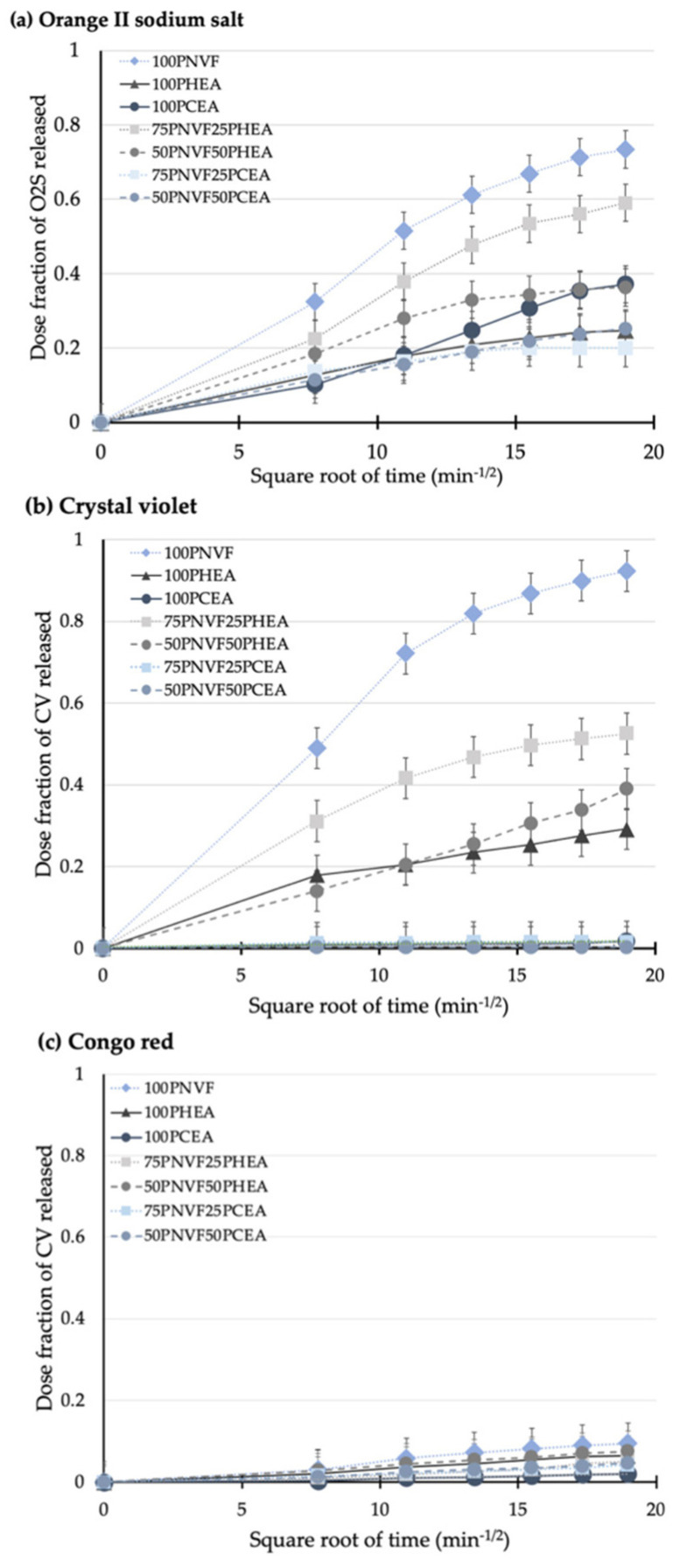
Release profiles of the hydrogel samples with dose fraction plotted vs. square root of time for (**a**) orange II sodium salt, (**b**) crystal violet, and (**c**) Congo red.

The correlation coefficients obtained from the various kinetic models are presented in Table 4. The data suggest that the release from the hydrogel samples studied was best described by Higuchi’s model. In order to further clarify the release kinetics, Figure 9a–c shows the Higuchi’s model curves for all samples. These finding suggests that the developed hydrogel matrices are effective in controlling release via a diffusion mechanism.

## 3. Conclusions

This work successfully produced a series of hydrogels based on NVF with two other monomers (HEA and CEA). The hydrogels were initially assessed for their water contact angles, %EWC, and water state ratios. The results show that all of the gels were hydrophilic and had water contents ranging from 52.4 to 94.6%. The homopolymer of NVF has the highest water content and a very high freezing or free water value. When calculating the amount of water molecules per NVF repeat unit, this value came to ~67 water molecules and a free to bound water ratio of 16.7 to 1. The copolymers gave a range of water contents and free to bound water ratios, with non-linear relationships existing between these properties, suggesting the possibility of composition-specific delivery systems. With the desired application and understanding to be the release of drug molecules or active agents from these hydrogels, a series of hydrophilic organic dye molecules were assessed. The uptake and release of dyes examined by color parameter of the hydrogels show that ΔE values for release agreed with the cumulative release profiles based on the relevant calibration curves of each dye. The diffusion-based release study found that the release of the dye molecules from the developed hydrogel matrices decreases over time and can be best described by Higuchi’s model. The empirical data suggest that the hydrogel’s capacity to release the dye is primarily determined by the presence of free water molecules within the hydrogel. However, certain combinations of dye and polymer can establish strong intermolecular interactions that supersede the influence of free water and become the prevailing factor in the hydrogel’s dye release behavior. Thus, when selecting polymer hydrogel materials for delivery applications, not only the structure of the gel should be considered, but also the free water in the structure to obtain the maximum release performance.

## 4. Materials and Methods

### 4.1. Materials

The following monomers were used in this study: N-vinylformamide (NVF) (98%), *N*-hydroxyethyl acrylamide (HEA), and 2-carboxyethyl acrylate (CEA). The crosslinker used throughout this study was poly(ethylene glycol) diacrylate (PEGDA; MW = 575) and the initiator was diphenyl (2,4,6-trimethylbenzoyl) phosphine oxide (TPO). For the drug release procedure, the following organic dyes were used: orange II sodium salt, crystal violet, and Congo red. All chemical used in this work were purchased from Sigma-Aldrich Co. Inc., Singapore.

### 4.2. Synthesis of Homo- and Copolymers Hydrogels Based on NVF

Hydrogels for this study were prepared using free-radical polymerization using the photo-initiator (diphenyl (2,4,6-trimethylbenzoyl) phosphine oxide (TPO)) and crosslinker poly (ethylene glycol) diacrylate (PEGDA). N-vinyl formamide (NVF), N-hydroxyethyl acrylamide (HEA), and 2-carboxyethyl acrylate (CEA) were used as the monomers. Table 1 shows the compositions of homo- and copolymers hydrogels based on NVF hydrogels fabricated via photopolymerization. The dry dimensions of the hydrogel were controlled by injecting 1.5 mL of the hydrogel mixture between two glass plates lined with PET sheets to prevent adhesion after polymerization. The mold size had a width of 4 cm, a height of 4 cm, and a thickness of 0.1 cm. The mold was then sealed with clips and placed under UVLEDs for 2 min to cure the mixture into a crosslinked polymer network. The polymer sheets were then removed from the mold and rinsed with de-ionized water to remove any unreacted monomer. The swollen hydrogel was then soaked in de-ionized water for 5 days with the water being changed every day before characterization.

### 4.3. Equilibrium Water Content

Equilibrium water content (%EWC) measurements were conducted using a gravimetric technique. First, the hydrogels were dehydrated by placing them in an oven at 60 °C for 72 h, after which the dehydrated gels were weighed and then replaced in the oven until the samples’ weight reached a constant value. Each hydrogel was measured three times under the same conditions and the average values were reported (n = 3). The equilibrium water content was calculated according to the formula in Equation (1):

%EWC = ((W_w_ − W_d_)/W_w_) × 100% (1)

where W_w_ and W_d_ are the hydrated and dehydrated weights of the hydrogel, respectively.

### 4.4. Differential Scanning Calorimetry (DSC)

The water structure in the hydrogel samples was measured using differential scanning calorimetry (DSC) (Mettler Model DSC1, Greifensee, Switzerland). This determined the percentage of freezing water in the hydrogels. The DSC heating curves of all hydrogel samples display endothermic peaks between the 19 and 21 min mark (−3 to 18 °C) owing to the melting of the various forms of water, which freeze during the cooling stage [30]. To prepare the samples, hydrated hydrogels were cut and weighed (~10 mg) and then sealed in an aluminum pan to prevent water evaporation; the process was repeated for each sample in triplicate (N = 3). The pan was then placed in the sample holder of the thermal analyzer. The samples were scanned using the following parameters:

Cool from 25 °C to −70 °C
Hold for 5 min at −70 °C
Heat from −70 °C to −25 °C at 20 °C/min
Heat from −25 °C to 25 °C at 10 °C/min

The area under the endothermic peak(s) corresponds to the energy required to melt the freezing water in the sample. The weight of the sample is known, as is the energy required to melt 1 g of pure water (333.5 Jg^−1^) [31]; thus, using Equation (2), we can calculate the percentage of freezable (free) water in the sample.

Free water (%) = [ΔHTr/(m × ΔHf)] × 100(2)

where ΔH_f_ = 333.5 J/g, ΔH_Tr_ = heat of transition, and m = sample weight (mg). The amount of bound water was obtained by subtracting the amount of freezing water from the total percent water content [32], whereby

Bound water content (%) = Total water content (%) − Free water content (%) (3)

### 4.5. Contact Angle (CA)

The wettability of the hydrated hydrogel surfaces was measured by contact angle (CA) using a Dataphysics Model OCA20 (Filderstadt, Germany) at room temperature. The hydrated hydrogel samples were cut into 2 cm^2^ pieces and placed onto a glass cover slide. A volume of 10 μL deionized water was dropped onto the surface sample with a micrometric syringe. A static image was taken and the contact angle was calculated using the built-in software. Each sample was tested 10 times (n = 10) and the average values were reported.

### 4.6. Dye Uptake and Release

For uptake and release studies, three organic dyes were used: orange II sodium salt dye, crystal violet dye, and Congo red dye. Dye uptake was carried out by immersing hydrated hydrogel samples into a solution containing a known concentration of dye. Each hydrogel was weighed and then soaked in 1.0 mL of dye solution with a 0.0001 M concentration of dye for 96 h to give time for the system to reach equilibrium before testing the dye release. To assess the capacity of each hydrogel to absorb dye, we measured the UV absorption of the dye solution both prior to and after the uptake process, using the calibration curves presented in Figure S1.

For the release study, each sample used the following method. After the uptake period (96 h), the hydrogels with the absorbed dye were placed into a new clean vial, which contained 1 mL of DI water. After transferring the hydrogel, the vial was vortexed using a Vortex Mixer GENIE 2 (Model G560E, Bohemia, NY, USA) at 5000 rpm for 15 s. The gel was then left in the solution for a set time. The study involved six 60 min time periods, which combined to make a total release time of 6 h. After each time period, the solution was removed and fresh media (1 mL DI water) was placed into the vial for the next time period. The amount of dye released was determined by measuring in triplicate (n = 3) the UV absorbance of the solution using a multiplate reader (Biotek, Model Synergy H1 Hybrid Reader Santa Clara, CA, USA). When using orange II sodium salt, a wavelength of 480 nm was used; for crystal violet, a wavelength of 590 nm was used; and for Congo red, a wavelength of 495 nm was used. The absorbance value at the specific wavelength for the given dye was then converted into a dose fraction using calibration curves of the appropriate dye.

## Figures and Tables

**Table 1:** Compositions of homo- and copolymers hydrogels based on NVF.

Sample Code	Main Monomer (%*w*/*w*)	Co-Monomer (%*w*/*w*)		CrossLinker (%*w*/*w* of Monomer)	Photo-Initiator (%*w*/*w* of Monomer)
	NVF	HEA	CEA		
100PNVF	100	-	-	3	1
100PHEA	-	100	-	3	1
100PCEA	-	-	100	3	1
75PNVF25PHEA	75	25	-	3	1
50PNVF50PHEA	50	50	-	3	1
75PNVF25PCEA	75	-	25	3	1
50PNVF50PCEA	50	-	50	3	1

**Table 2:** %EWC and free to bound water ratio of the hydrogel samples.

Composition	%EWC	Free to Bound Water Ratio
100PNVF	94.57	16.7:1
100PHEA	80.80	4.5:1
100PCEA	52.42	1.3:1
75PNVF25PHEA	85.80	7.4:1
50PNVF50PHEA	83.55	5.3:1
75PNVF25PCEA	80.78	3.9:1
50PNVF50PCEA	71.71	2.1:1

**Table 3:** Information of selected organic dyes.

Name	Dye Type	MW	pKa	Log *p* *
Orange II sodium salt	Anionic azo dye	350.32	8.26, 11.4	−0.95
Crystal violet	Cationic dye	407.99	9.4	1.17
Congo red	neutral-ionic azo dye	696.68	4.1	2.63

* Log *p*-values obtained from https://www.carlroth.com/ (accessed on 29 March 2023).

**Table 4:** Linear correlation obtained from different kinetic models.

Composition	Linear Correlation (R^2^)		
	Zero-Order	First-Order	Higuchi
O2S released			
100PNVF	0.8316	0.5845	0.9790
100PHEA	0.8015	0.6420	0.9731
100PCEA	0.9612	0.8070	0.9755
75PNVF25PHEA	0.8706	0.6406	0.9844
50PNVF50PHEA	0.7598	0.5858	0.9500
75PNVF25PCEA	0.6560	0.5465	0.8962
50PNVF50PCEA	0.8781	0.7097	0.9963
CV released			
100PNVF	0.7525	0.4671	0.9491
100PHEA	0.7649	0.5662	0.9538
100PCEA	0.7830	N/A	0.8550
75PNVF25PHEA	0.7209	0.5162	0.9354
50PNVF50PHEA	0.9430	0.7167	0.9954
75PNVF25PCEA	0.5175	N/A	0.7613
50PNVF50PCEA	0.4982	N/A	0.6590
CR released			
100PNVF	0.9063	0.8192	0.9835
100PHEA	0.9316	0.9014	0.9869
100PCEA	0.9639	0.7331	0.9394
75PNVF25PHEA	0.9876	0.9112	0.9103
50PNVF50PHEA	0.9184	0.8591	0.9976
75PNVF25PCEA	0.9482	0.8732	0.9762
50PNVF50PCEA	0.9448	0.9812	0.9866

## Data Availability

The raw/processed data required to reproduce these findings cannot be shared at this time as the data also forms part of an ongoing study.

## Supplementary Materials

The following supporting information can be downloaded at: https://www.mdpi.com/article/10.3390/gels9040333/s1, Table S1: Reproducibility and homogeneity of the prepared hydrogel samples (Dimensions and weights) Figure S1: Standard calibration curve of (a) orange II sodium salt, (b) crystal violet and (c) congo red dye solution at pH 7 in depended on the absorbance measurement.
