# Supplementary material for: Homo- and Copolymer Hydrogels Based on N-Vinylformamide: An Investigation of the Impact of Water Structure on Controlled Release
Source: Gels. 2023 Apr 14;9(4):333. doi: 10.3390/gels9040333 (PMC10138162; doi:10.3390/gels9040333)
Supplement: Supplementary file 1 [file gels-09-00333-s001.zip › gels-2309551-supplementary.pdf]

Supplementary Table and Figure

Table S1: Reproducibility and homogeneity of the prepared hydrogel samples (Dimensions and weights)

samples	n	Diameter (mm)	Thickness (mm)	Weight (g)
100PNVF	1	11.0	0.69	0.130
	2	11.0	0.70	0.132
	3	11.0	0.69	0.131
100PHEA	1	11.0	0.64	0.113
	2	11.0	0.64	0.114
	3	11.0	0.63	0.111
100PCEA	1	11.0	0.61	0.087
	2	11.0	0.62	0.092
	3	11.0	0.61	0.084
75PNVF25PHEA	1	11.0	0.68	0.132
	2	11.0	0.67	0.127
	3	11.0	0.67	0.124
50PNVF50PHEA	1	11.0	0.68	0.129
	2	11.0	0.68	0.129
	3	11.0	0.69	0.132
75PNVF25PCEA	1	11.0	0.62	0.101
	2	11.0	0.62	0.101
	3	11.0	0.62	0.102
50PNVF50PCEA	1	11.0	0.64	0.115
	2	11.0	0.64	0.115
	3	11.0	0.65	0.107

Figure S1: Standard calibration curve of dye solution

(a) Orange II sodium salt dye solution

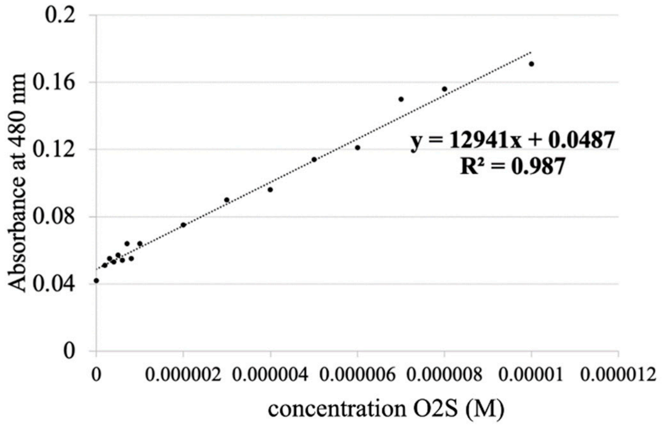

(b) Crystal violet dye solution

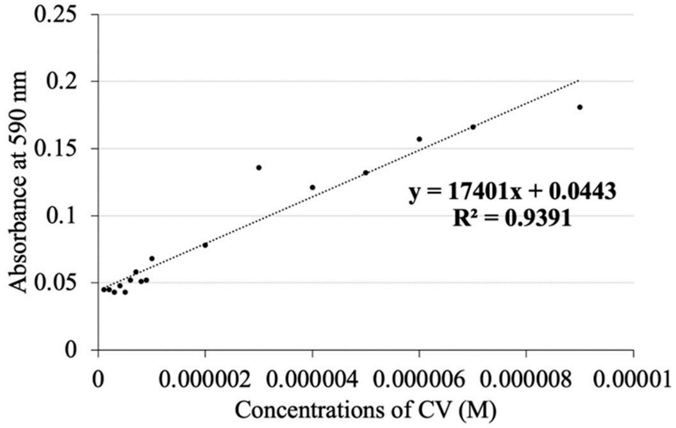

(c) Congo red dye solution

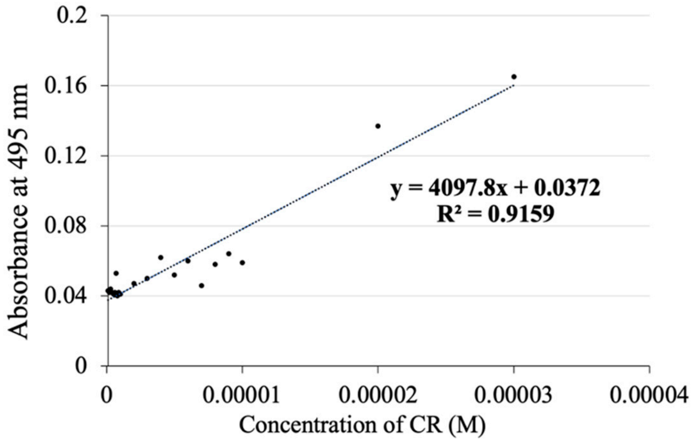

Figure S1. Standard calibration curve of (a) orange II sodium salt, (b) crystal violet and (c) congo red dye solution at pH 7 in depended on the absorbance measurement.
